# A Qualitative Study to Examine Perceptions and Barriers to Appropriate Gestational Weight Gain among Participants in the Special Supplemental Nutrition Program for Women Infants and Children Program

**DOI:** 10.1155/2016/4569742

**Published:** 2016-06-15

**Authors:** Loan Pham Kim, Maria Koleilat, Shannon E. Whaley

**Affiliations:** ^1^Pepperdine University, Malibu, CA 90263, USA; ^2^California State University, Fullerton, CA 92831, USA; ^3^PHFE-WIC Program, Irwindale, CA 91706, USA

## Abstract

Women of reproductive age are particularly at risk of obesity because of excessive gestational weight gain (GWG) and postpartum weight retention, resulting in poor health outcomes for both mothers and infants. The purpose of this qualitative study was to examine perceptions and barriers to GWG among low-income women in the WIC program to inform the development of an intervention study. Eleven focus groups were conducted and stratified by ethnicity, and each group included women of varying age, parity, and prepregnancy BMI ranges. Participants reported receiving pressure from spouse and family members to “eat for two” among multiple barriers to appropriate weight gain during pregnancy. Participants were concerned about gaining too much weight but had minimal knowledge of weight gain goals during pregnancy. Receiving regular weight monitoring was reported, but participants had inconsistent discussions about weight gain with healthcare providers. Most were not aware of the IOM guidelines nor the fact that gestational weight gain goals differed by prepregnancy weight status. Results of these focus groups analyses informed the design of a pregnancy weight tracker and accompanying educational handout for use in an intervention study. These findings suggest an important opportunity for GWG education in all settings where pregnant women are seen.

## 1. Introduction

The current high rates of obesity continue to be a significant public health concern [1]. The childbearing years pose potential obesity risk for women because of excessive gestational weight gain (GWG) and postpartum weight retention [2–4]. Excess GWG and postpartum weight retention result in suboptimal pre- and perinatal health and predispose both mothers and their infants to long-term chronic diseases. In response to the mounting evidence, the Institute of Medicine (IOM) revised its 1990 recommendations and released new GWG recommendations in 2009 [5, 6]. These guidelines are specific to a woman's prepregnancy body mass index (BMI); for example, underweight women (BMI of <18.5 kg/m^2^) should gain 28–40 lbs; normal weight women (BMI of 18.5–24.9 kg/m^2^) should gain 25–35 lbs; overweight women (BMI of 25.0–29.9 kg/m^2^) should gain 15–25 lbs; and obese women (BMI ≥ 30.0 kg/m^2^) should gain 11–20 lbs. Despite these guidelines, almost one-half of women exceeded the IOM recommendations [7]; groups particularly at risk for excess GWG are Black and Hispanic mothers [8, 9]. Adverse health outcomes from excess GWG during pregnancy include gestational diabetes, hypertensive disorders, cesarean delivery and operative complications [10, 11], and postpartum overweight and obesity [2–4, 12]. Excessive GWG from a previous pregnancy is a significant nutritional risk factor for subsequent pregnancies, leading to adverse health outcomes for the child, including increased risk of macrosomia and childhood obesity [10, 13, 14]. Consequently, increasing trends of maternal overweight and excessive GWG may set the stage for a transgenerational cycle of obesity as heavier mothers give birth to heavier daughters, who are at increased risk of becoming obese themselves during their childbearing years [15].

The Special Supplemental Nutrition Program for Women Infants and Children (WIC) is a food and nutrition education program for pregnant, breastfeeding, and postpartum women, infants, and children under age five who are low-income (up to 185% of the Federal Poverty Level) and at nutritional risk. Nationwide, approximately 25% of the individuals served are women, and approximately half of these women are pregnant with the other half postpartum. WIC services are available in every state and US territory, and currently WIC services are delivered to over 8 million participants each month. In Los Angeles County (LAC), WIC currently serves approximately 67% of all infants and about half of all children ages one to five, translating into approximately 500,000 individuals and 350,000 families each month. Because of WIC's deep reach in low-income ethnic minority communities, understanding and programming interventions to target WIC participants can potentially lead to significant positive outcomes in perinatal health of women and their young children.

Because WIC is uniquely positioned to potentially mediate perinatal health of mothers and children, especially ethnic minority and underresourced communities, research on GWG in this population is vital. The most recent quantitative studies of GWG have focused on predictors of child weight based on maternal weight [16, 17]; only two studies have specifically focused on women in the WIC program [18, 19]. Qualitative studies have the advantage of allowing researchers to explore GWG in multiple and complex dimensions such as knowledge, attitudes, opinions, and reported behaviors during pregnancy. Currently, the qualitative literature has a scant, but growing body of work which document knowledge and barriers to gestational weight gain, and most of these studies have focused on low-income Black or Hispanic women [20–22]. The results of these studies suggest the need for more qualitative work in these communities to better understand cultural barriers and facilitators of appropriate GWG during pregnancy.

One qualitative study of African-American women suggested focusing research on GWG in the WIC program in order to develop strategies for improving long-term health of mothers and their children [22]. To date, we are not aware of any qualitative studies which have specifically examined the perceptions of GWG among an ethnically diverse group of participants in the WIC program. Therefore, the objectives of this qualitative study were threefold: (1) to explore knowledge, attitude, and perceptions regarding weight gain during pregnancy among participants in the WIC program; (2) to assess participants' knowledge and awareness of the IOM guidelines for weight gain during pregnancy; and (3) to solicit participant feedback and suggestions to inform the development of a gestational weight gain intervention in the WIC program.

## 2. Materials and Methods

### 2.1. Study Design, Participants, and Recruitment

Between February and December 2013, we conducted a series of 11 focus group interviews with a total of 59 WIC participants across 3 ethnic groups (Caucasian, Black, and Hispanic) to inform the design a gestational weight gain intervention among WIC participants. These ethnic groups were chosen because they represent the largest ethnic groups in the US as well as in the WIC program [23, 24]. WIC participants were recruited for this study if they met the study criteria of being pregnant at the time of the study and self-identified as white/Caucasian, Black/African-American or Hispanic/Latina. Each focus group was stratified by ethnicity in order to explore potential cultural variations in perceptions, attitudes, and behaviors with regard to gestational weight gain. Within each focus group, there was diversity in age, parity, and prepregnancy BMI ranges among the participants. Focus groups with Hispanic participants were grouped into English-speaking and Spanish-speaking in order to facilitate discussion among those with limited English proficiency. Recruitment of focus group participants and facilitation of focus group interviews were accomplished with support from staff from the Public Health Foundation Enterprise (PHFE) WIC Program in a number of WIC centers around Los Angeles, California.

Inductive semistructured focus group guides were developed by the research team, with feedback from PHFE-WIC research staff. Open-ended questions were utilized to stimulate discussion and included probes to address more specific dimensions of each topic. Focus group guides explored the following major topics: (1) health perceptions during pregnancy; (2) barriers to appropriate weight gain; (3) weight monitoring during pregnancy; (4) knowledge and recognition of IOM weight gain guidelines. Additionally, we solicited participant opinions on how to develop educational tools, including a gestational weight gain tracker that would help mothers track their weight gain through pregnancy, for the intervention phase of the study. Focus group guides were translated into Spanish and back-translated to ensure integrity and consistency.

Focus group moderators were trained in qualitative research and had experience moderating focus group interviews. The Spanish-speaking focus group moderator was bilingual and bicultural. Focus groups ranged from 5 to 8 women, and each session lasted an average of 90 minutes, with question and answer format so the discourse was unhurried. Prior to the start of a focus group discussion, participants provided written informed consent and completed a short demographic questionnaire which included self-reported height and prepregnancy weight. All focus group sessions were videotaped and audio recorded. All recording files were password protected and stored on a secure server at Pepperdine University. All transcripts were transcribed verbatim by an independent contractor; focus group sessions conducted in Spanish were transcribed verbatim in Spanish, then translated to English, and checked for accuracy by an independent reviewer. All aspects of the study were reviewed and approved by the University of California, Los Angeles, and Pepperdine University's Institutional Review Board for the protection of human research subjects. As a token of gratitude for their time and participation in the study, each participant received a $20 gift card.

### 2.2. Data Analysis

Descriptive statistics were computed using SPSS version 22 (Chicago, IL) and are summarized in Table 1. All transcripts and interviewer notes were organized and coded using ATLAS.ti version 6.1 (ATLAS.ti Scientific Software Development GmbH, Berlin, Germany) and analyzed using thematic analysis [25, 26]. The research team, consisting of the lead author and research students trained in qualitative methods, analyzed these data. The analysis process began with the research team conducting multiple independent readings of the transcripts to allow themes to emerge. To develop consensus, the research team convened after the initial reading to discuss codes and build emerging themes. Subsequently, a second pass of the transcripts was completed to ensure that all themes were captured. Following a third reading, the research team convened again to discuss any additional themes and reconciled any inconsistencies. Percentages presented in the results section reflect participant responses during the focus group by a show of hands or a nod in response to a count question. The researchers compiled these percentages by reviewing the video recordings to count participant responses.

## 3. Results and Discussion

### 3.1. Participant Characteristics

Demographic characteristics are summarized in Table 1. Focus groups were stratified by ethnicity, and overall about one-fifth of the participants were Caucasian, one-third were Black, and nearly half were Hispanic. On average, focus group participants were 28 years old and had been in the WIC program for 2.39 years, and about half (50.8%) of the participants were pregnant with their first child. More than half of the participants were born outside of the US and of these, most reported being from Mexico or Central America. About one-third of the participants were married, and more than 75% of the participants had at least a high school diploma or GED equivalent. More than half of the participants were either overweight or obese.

### 3.2. Focus Group Concepts and Emerging Themes

 The following shows sample focus group questions organized by the four major topics.

 Perceptions of health during pregnancy:

 To you, what does it mean to be healthy?
 Generally, how healthy do you think you are? Would you say you are in excellent, very good, fair or poor health? Why?
 During pregnancy, do you think you are as healthy as when you are not pregnant? Why?

Barriers to appropriate GWG:

 How would you say you are doing in terms of weight gain during pregnancy? Would you say you are gaining too, too little, or just the right amount of weight?
 Are you concerned about how much weight you have gained at this time? Does anyone in your family share your concerns about how much weight you have gained?
 What is the biggest challenge for you to gain the right amount of weight during pregnancy?
 What do you think are the consequences of gaining too much or too little weight during pregnancy?

Weight monitoring during pregnancy:

 Are you weighing yourself or getting weighed regularly?
 When you come for your pre-natal visits, does your doctor talk with you about how much weight you have gained? Please share about this experience.
 When you come for your regular WIC visit, do WIC staff talk with you about your weight? Please share about this experience.
 When WIC staff talk with pregnant moms about their weight, how do you think WIC staff should approach the topic of weight gain during pregnancy?

Knowledge and recognition of IOM guidelines and ideas for education:

 Have you heard of or seen these IOM guidelines for weight gain?
 Are you aware there are different weight gain recommendations for pregnant women, based on your pre-pregnancy weight? If so, where did you learn about these recommendations?
 When we develop the GWG tracker tool [participants provided samples], how should we differentiate the different pre-pregnancy weight groups? Should we put these terms (underweight, normal weight, overweight or obese) on the tracker?
 If WIC develops classes about gaining the right amount of weight, what specific issues do you think we need to address?

**Figure 1:**
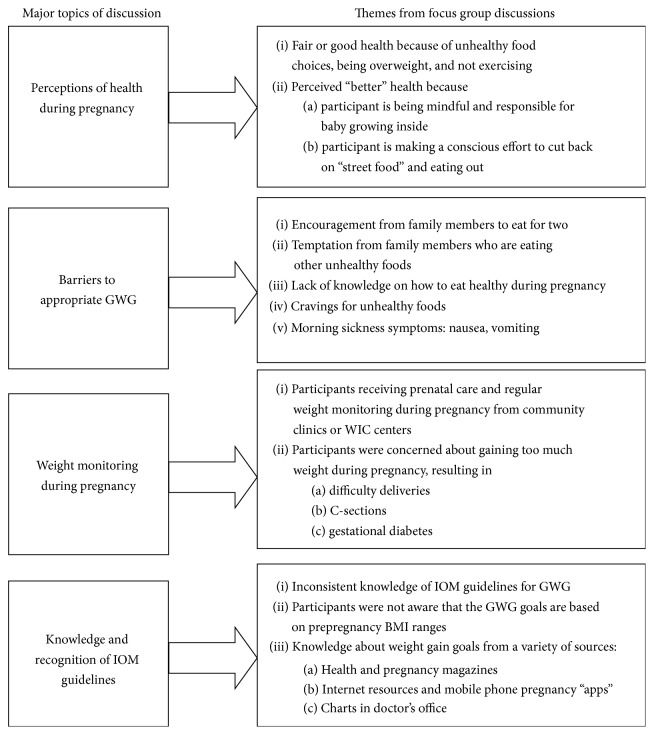
Overview of focus group topics and emerging themes.

Figure 1 provides a schematic overview of the topics discussed in the focus groups; within each topic, clear themes emerged from focus groups discussions, and we describe them in more detail below.

#### 3.2.1. Perceptions of Health during Pregnancy

The focus group discussion began with a general open-ended question about participants' perception of health. Participants were asked to rate their health as excellent, good, fair, or poor and to elaborate on why they chose a particular rating. A majority of the participants (85%) rated their health as either fair or good; only 4 of the 59 participants reported being in excellent health. Those participants who indicated having fair or good health commented that they rated their health that way because they were overweight or ate too much fast food or junk food. For those who indicated that they were in poor health, they also reported being overweight, not exercising, and eating out or eating “street food” too often. For the few who indicated being in excellent health, they reported eating healthy, exercising, and drinking water on a regular basis.

When asked about their perception of health during pregnancy, a majority of the participants (more than 80%) indicated that they felt healthier during pregnancy than when they were not pregnant. When probed about why they felt healthier, participants reported (1) being mindful of the baby inside and feeling responsible to help baby grow as healthy as possible and (2) trying to choose healthier foods, cutting back on “street food” and cooking at home, consuming less junk food, drinking more water, and cutting back on sodas and other sugar-sweetened beverages. One mother shared, “I think I'm healthier because I feel like I've got to do this for the baby more than me.” Another participant stated, “I would say [I am] more healthy because you have the responsibility to take care of yourself and your baby who's growing inside of you. You want a healthy baby.”

#### 3.2.2. Barriers to Healthy Weight Gain during Pregnancy

When asked about their ability to maintain appropriate and healthy weight gain during pregnancy, participants reported the following reasons as barriers to being able to maintain healthy weight, which these women defined as “not too much, not too little weight gain.” The first barrier was the encouragement from family members to “eat for two.” Participants shared that spouses, mothers-in-law, siblings, and grandparents encouraged them to eat more because they had to feed their babies. As a result, these women reported difficulty in being able to maintain their weight. Even when they did not want to eat, participants reported being strongly encouraged to eat more by family members. Second, participants reported having cravings as a barrier to being able to maintain healthy weight, as one mother reported, “With my first son, I craved a lot of fruit, so it was more healthy. But this time around, I crave chocolate, cakes, all that sugary stuff.” Third, participants indicated that being tempted by what family members were eating around them was another barrier to maintaining healthy weight. Because the family members were able to eat without any restrictions, these pregnant women also wanted to be able to eat whatever others around them were eating. Many participants were cognizant that they could not because of the excessive calories associated with overeating, particularly high sugar foods. One participant lamented, “It's harder because I have my kids and my husband. They want to go get burgers and here I am with my turkey sandwich. If it was my first pregnancy, it [wouldn't] be as hard. It's harder because my kids want soda. I don't give them that much, but sometimes just a cup of soda. I want some soda too but I can't. It's harder on me because of my family.” Another barrier reported was the lack of knowledge about what to eat during pregnancy. One participant shared, “When I lived with my mother-in-law, she would eat a lot of meats, but also she would cook a lot of vegetables – boiled. So now that I live on my own, I look in the refrigerator and I'm like, ‘well, what do I cook?' I know how to cook, but I just don't know, what [do I] cook? Or when I'm in there, or I look at the pantry and I'm like ‘how do I make that?'”. The last barrier reported was having morning sickness symptoms such as nausea and vomiting as barriers to being able to eat healthy.

#### 3.2.3. Weight Monitoring during Pregnancy

When the discussion focused on weight monitoring during pregnancy, all participants shared affirmatively that they were concerned about their weight, but they did not know how much weight they should be gaining during pregnancy. These concerns centered on having difficult deliveries or complications, such as C-sections. One mother shared, “I'm actually very concerned if I gain a lot of weight because my doctor said if I gain a lot more than I should, it will be very hard for the delivery time.” Second, women were concerned about weight loss after delivery. One mother reported, “Gaining too much. It's hard to lose the weight after. You have to think of the aftermath because once the baby comes out you're stuck with this weight.” Finally, about one-third of the women shared that they were concerned about developing gestational diabetes if they were overweight during pregnancy. One mother suggested, “I guess, if you gain too much weight, then your baby is going to be bigger and then in my case if I gain too much, then I can get gestational diabetes, which I never had with any of my other pregnancies.”

All women reported receiving some form of prenatal care and weight monitoring during their pregnancy, either from a private doctor or community clinic. Most women also reported being weighed regularly at a prenatal visit or at WIC. Additionally, some participants also weighed themselves regularly at home; these participants were particularly concerned about their risk of developing gestational diabetes. A few women admitted to being in denial, not wanting to know their weight. Some felt that pregnancy was a “special time” in their lives, so weight should not be the focus. Others were resigned that they were already “big,” so it was of no benefit to step on a scale. One participant lamented, “I don't [weigh myself] at home. I don't know, I just think that if I do it I'm going to obsess over the weight, and I don't want to harm my baby. But then if I feel that my clothes don't fit, I'll start drinking water.”

When asked if they discussed weight gain goals during pregnancy with their healthcare providers during prenatal visits, participants shared inconsistencies with regard to whether their doctors discussed weight gain during pregnancy. For some participants, their doctors discussed weight gain goals and provided feedback so participants were aware of whether they were gaining the appropriate amount of weight during pregnancy. For other participants, their doctors told them they were “fine” and did not have to worry about their weight. Some others shared that there was minimal or no discussion of their weight during their monthly prenatal visits. One participant reported, “In my case, they haven't told me that I am gaining too much weight. They always tell me that I am fine and that is why I don't worry. Now if they told me that I gained too much then I would have to do something; but till now they haven't told me that I am bad.”

#### 3.2.4. IOM Guidelines for Weight Gain during Pregnancy

A few women (5 of 59) had no knowledge or awareness of any guidelines for weight gain during pregnancy; most of these women were more recent immigrants. Some participants had seen weight gain goals and were aware of the concept of weight gain goals during pregnancy. Among those who knew about weight gain ranges, there were inconsistencies with the weight gain ranges reported by these participants. Some reported ranges that were higher than the IOM guidelines, while others reported lower weight ranges. Among those who knew about weight gain ranges, some shared that they knew about weight gain goals from their doctor, charts in the doctor's office, educational pamphlets, baby magazine and “baby” books, or online resources (websites and mobile phone applications or “apps”). A few women indicated they had learned about their weight gain goals from WIC staff. One participant reported, “Here in WIC, yes. Every time they weigh me they say that 10–15 pounds is what is normal to gain during pregnancy for me. But in the [health] clinic, no.” Most importantly, most women (>75%) were not aware that their gestational weight gain goals differed by prepregnancy weight status. These results are confirmed by Anderson and colleagues, who also found that women received inadequate or conflicting information about pregnancy nutrition and GWG from health care providers [27]. This suggests an important opportunity and avenue for education in all settings where pregnant women are routinely seen.

#### 3.2.5. Ideas for Educational Tools: A Pregnancy Weight Tracker

One of the complications of determining appropriate GWG is that guidelines are based on four categories of prepregnancy weight: underweight, normal weight, overweight, or obese. In the focus group discussions of how to differentiate the pregnancy weight tracker tool based on prepregnancy weight status, participants offered different ideas for how to identify the woman's weight category. Some participants wanted the weight designation (underweight, normal weight, overweight, and obese) listed on the pregnancy weight tracker because it was an accurate reflection of their weight status; others expressed concern that the labels posed a negative stigma. One mother said, “I agree, some people might not be able to accept that [they are overweight]. I don't know, but I agree you shouldn't get mad, because it's the truth if you're overweight.” For a majority of Caucasian and Hispanic participants, the designation of “overweight” or “obese” on the tracker was a sensitive issue, and they preferred not to have these labels on the pregnancy weight tracker; instead, the participants suggested color coding the four weight ranges and including the weight range goals on the pregnancy weight tracker. One participant commented, “You don't know how sensitive a person may be when it comes to words like ‘obese' or ‘overweight', and like she said, what does it mean? How do you define that for a person? So, I think maybe that's a bit more sensitive matter.” Another participant suggested distinguishing the different weight categories by color: “No, I don't think [overweight or obese] should be on the pregnancy weight tracker. I think it would be a constant reminder that you're overweight. But having different colors for different weights is better.”

When asked if participants were interested in having a way to track their weight gain during pregnancy, all the participants indicated a strong interest in having a tool to track their weight during pregnancy. Most women reported a willingness to use a pregnancy weight tracker if WIC provided one; also, the women suggested that the pregnancy weight tracker would be helpful to facilitate discussion of their weight gain goals at regular prenatal visits with their healthcare provider, as well as WIC visits. A majority of the women indicated that they would like a “credit card” sized pregnancy weight tracker for their purse and “half sheet” to be used at home, to be placed either on the refrigerator or in the bathroom. Participants shared affirmatively that they were interested in having accountability with using the pregnancy weight tracker, along with educational handouts. Participants shared that having a pregnancy weight tracker (GWG tracker) on hand to discuss with WIC staff and their doctors would encourage them to monitor their own weight and stay on track with weight gain goals. One participant suggested that the pregnancy weight tracker should be a routine part of WIC services, “They ask about our address and income when we come, and they should ask about that [pregnancy weight tracker] too when they give it out.”

#### 3.2.6. Applications of Focus Group Findings to an Intervention Study

Findings from this study suggest that participants face significant barriers to being able to gain the appropriate amount of weight and are very interested in tools and education to help stay on target with weight gain. Results of focus groups analyses were utilized to design a GWG tracker and accompanying educational handout for use with pregnant moms enrolling in WIC. A GWG tracker was developed based on their feedback (compact size so participants can carry it with them and color coded rather than identifying their weight status). The educational handout incorporated messages related to the barriers women identified. The first barrier discussed by the participants was the theme of “eating for two.” This perception of having to “eat for two” contributed to participants feeling the need to eat more, which then encouraged higher gestational weight gain rather than discouraging it. These findings are confirmed in other qualitative research studies with African-American and Puerto Rican mothers [20, 21, 28]. This suggestion from family members to eat for two is derived from motivation and desire to optimize pregnancy outcomes and fetal growth. If mothers do not perceive excess weight gain to be problematic, there will be little “buy-in” to follow the IOM guidelines. These perceptions of threat and susceptibility are at the heart of the Health Belief Model [29]; as such, these findings suggested the need to direct nutrition education to target debunking this myth of “eating for two.” The educational handout developed for the intervention phase served to debunk the myth in the following ways: (1) providing clarification for weight gain distribution during pregnancy; (2) explaining how excess weight gain leads to complications with pregnancy and delivery; (3) providing IOM weight gain ranges so participants are aware and can track their weights during pregnancy.

**Figure 2:**
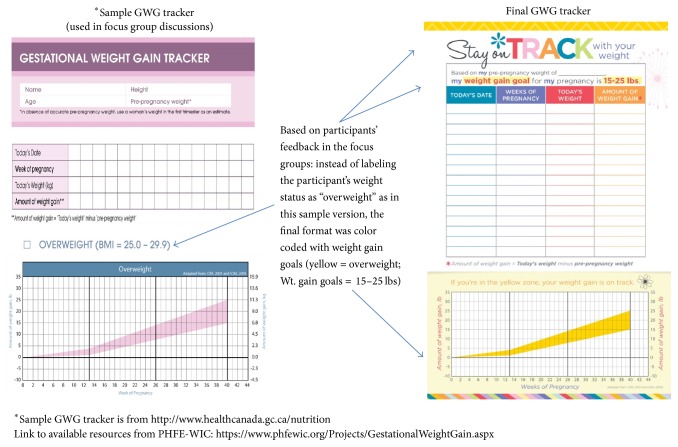
Sample and final pregnancy weight tracker (GWG tracker).

We found that a majority of the focus group participants were concerned about gaining too much weight but had many misperceptions and barriers that needed to be clarified and corrected; these findings are corroborated by another study [22]. The educational handout serves to debunk myths and correct understandings about GWG.  Figure 2 provides a sample GWG tracker tool that was developed for use during the focus group discussions; focus group participant feedback and suggestions led to the final GWG tracker. This GWG tracker tool allows participants to be accountable about their weight gain goals and facilitates discussions with their doctors about weight gain during prenatal visits and with WIC nutritionists. Finally, placement of the GWG goals on the pregnancy weight tracker builds awareness among participants of appropriate weight gain during their pregnancy. Building this awareness among WIC participants for appropriate GWG provides important perceived benefits which, based on the Health Belief Model [29, 30], may go a long way in raising consciousness among WIC participants.

While this qualitative study provided important grounding for the development of an intervention study to prevent excessive GWG among WIC participants, it is not without its limitations. Because the sample is specific to the WIC population, these findings may have limited generalizability to the entire population of pregnant women who have varying socioeconomic and cultural backgrounds. Second, the Hispanic population of WIC participants is primarily from Mexico, and this may not be generalizable to WIC programs in other parts of the US. Finally, while it is possible that our findings may have been impacted by the presence of women with different BMI classifications within a focus group, our moderators used qualitative methodology skills to encourage all participants to share. It would be beneficial in future studies to also further stratify focus groups by BMI classifications to ensure a more homogenous grouping of women based on weight groupings. Despite these limitations, the benefit of this qualitative study was to allow for an unhurried and open discussion of the IOM gestational weight gain guidelines and challenges to appropriate weight gain during pregnancy in the WIC program; the findings from these discussions were applied directly to inform the development of the intervention.

## 4. Conclusion

Findings from this study have important implications for how perinatal health is conceptualized and assessed. These findings illustrate the complex nature of weight gain during pregnancy, particularly among low-income ethnic minority groups. This work extends the discussion beyond a simplistic and unidimensional conceptualization to one which considers the multilevel and complex socioecological landscape in which immigrant communities navigate their health decisions. Employing qualitative research methods in maternal and child health research with immigrant groups allow for findings and interpretations to be grounded in the cultural context of daily lived experiences. Second, narratives give voice to and illuminate our understanding of how individual experiences collectively and simultaneously affect pregnancy outcomes, thus providing a more holistic perspective on the impact of culture on perinatal health. At a practical level, this holistic approach allows for a better understanding of the contextual environment in which many of these low-income families live; as a result, we will be able to design WIC-based programming and health-related interventions that will address these barriers and move us forward in bridging the gap in health disparities.

## Figures and Tables

**Table 1:** Description of the sample (*N* = 59).

	*N*	%	Mean	s.d.
*Age*	59		28.20	5.41

*Years in WIC*	59		2.39	3.16

*Years in the US*	24		14.27	7.69

*Birthplace*				
US born	35	59.3		
Foreign born	24	40.7		

*Children in WIC*				
Pregnant	30	50.8		
One child	21	35.6		
Two children	8	13.6		

*Ethnicity*				
White	13	22.0		
Black	20	33.9		
Hispanic-English-speaking	18	30.5		
Hispanic-Spanish-speaking	8	13.6		

*Marital status*				
Married	19	32.2		
Divorced	3	5.1		
Separated	4	6.8		
Never married	33	55.9		

*Education*				
Up to 12th grade	16	27.1		
High school grad or GED	15	25.4		
Some college/associate	21	35.6		
Bachelors and beyond	7	11.9		

*Employment*				
Self-employed	17	28.8		
Unemployed	24	40.7		
Homemaker	9	15.2		
Student	6	10.2		
Unable to work	3	5.1		

*Maternal BMI*				
Underweight	4	6.8		
Normal weight	24	40.7		
Overweight	12	20.3		
Obese	19	32.2		
